# The 4G/4G Genotype of PAI-1 Polymorphism Is Associated with Higher Plasma PAI-1 Concentrations and Mortality in Patients with Severe Sepsis

**DOI:** 10.1371/journal.pone.0129565

**Published:** 2015-06-11

**Authors:** Leonardo Lorente, María M. Martín, Juan M. Borreguero-León, Ysamar Barrios, Jordi Solé-Violán, José Ferreres, Lorenzo Labarta, César Díaz, Alejandro Jiménez

**Affiliations:** 1 Intensive Care Unit, Hospital Universitario de Canarias, La Laguna, Santa Cruz de Tenerife, Spain; 2 Intensive Care Unit, Hospital Universitario Nuestra Señora Candelaria, Santa Cruz Tenerife, Spain; 3 Laboratory Deparment, Hospital Universitario de Canarias, La Laguna, Tenerife, Spain; 4 Laboratory Deparment of the Research Unit, Hospital Universitario de Canarias, La Laguna, Santa Cruz de Tenerife, Spain; 5 Intensive Care Unit, Hospital Universitario Dr. Negrín, Las Palmas de Gran Canaria, Spain; 6 Intensive Care Unit, Hospital Clínico Universitario de Valencia, Valencia, Spain; 7 Intensive Care Unit, Hospital San Jorge, Huesca, Spain; 8 Intensive Care Unit, Hospital Insular, Las Palmas de Gran Canaria, Spain; 9 Statistical Deparment of the Research Unit, Hospital Universitario de Canarias, La Laguna, Santa Cruz de Tenerife, Spain; University of Torino, ITALY

## Abstract

**Objective:**

Two studies have reported that patients with the 4G/4G genotype of the plasminogen activator inhibitor-1 (PAI-1) genetic polymorphism had higher plasma PAI-1 concentrations and higher risk of death than those with the 4G/5G or 5G/5G genotypes; one study involved 175 children with meningococcal disease, and the other included 88 adult patients with septic shock. Thus, the objective of this study was to determine whether there is an association between carriage of the 4G/4G genotype, plasma PAI-1 concentrations and mortality in a large series of adult septic patients.

**Methods:**

An observational, prospective, multicenter study was carried out in six Spanish Intensive Care Units including severe septic patients. We determined the PAI-1 4G/5G polymorphism and plasma PAI-1 concentrations in all patients. The end-points of the study were 30-day and 6-month mortality.

**Results:**

We included a total of 260 patients, 82 (31.5%) with 4G/4G, 126 (48.5%) with 4G/5G and 52 (20.0%) with 5G/5G genotype. Multivariate logistic regression analysis showed that the 4G/4G genotype was associated with higher mortality at 30 days (Odds Ratio = 1.95; 95% CI = 1.063–3.561; p = 0.03) and at 6 months (Odds Ratio = 2.19; 95% CI = 1.221–3.934; p = 0.01), and that higher plasma PAI-1 concentrations were associated with higher mortality at 30 days (Odds Ratio = 1.01; 95% CI = 1.002–1.022; p = 0.02) at 6 months (Odds Ratio = 1.01; 95% CI = 1.003–1.023; p = 0.01). Multivariate linear regression analysis showed that increased plasma PAI-1 concentrations were associated with the PAI-1 4G/4G genotype (regression coefficient = 4.82; 95% CI = 3.227 to 6.406; p<0.001).

**Conclusions:**

The major findings of our study, to our knowledge the largest series reporting data about 4G/5G polymorphism of the PAI-1 gene, plasma PAI-1 concentrations and mortality in septic patients, were that septic patients with the 4G/4G genotype had higher plasma PAI-1 concentrations and higher risk of death than those with 4G/5G or 5G/5G genotypes.

## Introduction

Sepsis represents a systemic response of the immune system to infection, which is a common, expensive, and frequently fatal condition. During sepsis, the pro-coagulant and anti-fibrinolytic pathways lead to microvascular fibrin deposition, resulting in multi-organ failure, and ultimately death. [1].

Plasminogen activator inhibitor-1 (PAI-1) plays an important role in the fibrinolytic response [2]. Previous studies have found higher plasma PAI-1 concentrations in non-surviving than in surviving septic patients [3–12]. In addition, the association between PAI-1 4G/5G polymorphism and mortality in septic patients has been studied [10–20]. Some authors report that patients with the 4G/4G genotype of the PAI-1 gene had higher mortality rates than patients with other genotypes [10–14]; however, this was not found in other studies [15–20]. A recently published meta-analysis [21], including most of those studies [10–19], found that patients with the 4G/4G genotype of the PAI-1 gene had a higher risk of death than patients with 4G/5G or 5G/5G genotypes. However, only 3 of those studies also measured plasma PAI-1 concentrations [10–12]. Two studies have reported that patients with the 4G/4G genotype of the PAI-1 gene had higher plasma PAI-1 concentrations and higher risk of death than those with the 4G/5G or 5G/5G genotypes; one study involved 175 children with meningococcal disease [10], and the other included 88 adult patients with septic shock [11]. Another study with 166 adult septic patients reported that patients with the 4G/4G genotype and that patients with higher plasma PAI-1 concentrations had a higher risk of death; however, an association between PAI-1 4G/5G polymorphism and plasma PAI-1 concentrations was not reported [12]. Thus, the objective of this study was to determine whether there is an association between carriage of the PAI-1 4G/4G genotype, plasma PAI-1 concentrations and mortality in a large series of adult septic patients.

## Materials and Methods

### Design and Subjects

A multicenter, cohort study was carried out in 260 patients with severe sepsis from six Spanish Intensive Care Units. The study was approved by the Institutional Review Boards of the six participating hospitals: Hospital Universitario de Canarias (La Laguna. Santa Cruz de Tenerife. Spain), Hospital Universitario Nuestra Señora de Candelaria (Santa Cruz de Tenerife. Spain), Hospital Universitario Dr. Negrín (Las Palmas de Gran Canaria. Spain), Hospital Clínico Universitario de Valencia (Valencia. Spain), Hospital San Jorge (Huesca. Spain) and Hospital Insular (Las Palmas de Gran Canaria. Spain). Written informed consent from the patients or from their family members was obtained.

The diagnosis of severe sepsis was established according to the International Sepsis Definitions Conference [22]. Exclusion criteria were: age <18 years, pregnancy, lactation, human immunodeficiency virus (HIV), white blood cell count <1,000/mm^3^, solid or hematologic tumour, or immunosuppressive, steroid or radiation therapy.

### Variables recorded

The following variables were recorded for each patient: age, sex, diabetes mellitus, ischemic heart disease, chronic obstructive pulmonary disease (COPD), Acute Physiology and Chronic Health Evaluation II (APACHE II) score [23], activated partial thromboplastin time (aPTT), empiric antimicrobial treatment, bilirubin, bloodstream infection, creatinine, international normalized ratio (INR), lactic acid, leukocytes, microorganism responsible, pressure of arterial oxygen/fraction inspired of oxygen (PaO_2_/FiO_2_), platelets, site of infection, and Sepsis-related Organ Failure Assessment [SOFA] score [24]. The end-points of the study were 30-day and 6-month mortality.

### Blood samples

Blood samples were collected from patients at the time severe sepsis was diagnosed. Venous blood samples were placed in citrated plasma tubes and centrifuged within 30 minutes at 1000g for 15 minutes, and frozen at -80°C until the determination of PAI-1 concentration. In addition, venous blood were placed in EDTA-containing tubes and frozen at -80°C until determination of genetic polymorphism of PAI-1.

### Gene analysis

Gene analysis was centralized at the Research Unit of the Hospital Universitario de Canarias (La Laguna. Tenerife. Spain). Venous blood samples in EDTA-containing tubes were subjected to DNA purification using proteinase K, phenol-chloroform extraction, and ethanol precipitation. Genotyping was performed in a blinded manner, without knowledge of any clinical data. PAI-1 4G/5G polymorphism was determined for all patients using primers and restriction endonuclease digestion. In addition, 22 neutral markers were genotyped to follow genomic control strategies that would detect spurious associations due to population substructure. The neutral markers chosen were Alu repeats distributed throughout the genome.

### Plasma PAI-1 level assay

The assay of plasma PAI-1 concentrations was centralized at the Laboratory Department of the Hospital Universitario de Canarias (La Laguna. Tenerife. Spain). PAI-1 antigen was assayed by specific ELISA (Imubind Plasma PAI-1 Elisa. American Diagnostica, Inc., Stanford, CT, USA) according to the manufacturer's instructions. The assay detects latent (inactive) and active forms of PAI-1 and PAI-1 complexes. The interassay coefficient of variation (CV) was <5% (n = 20) and the detection limit for the assay was 1 ng/ml.

### Statistical Methods

Continuous variables are reported as medians and interquartile ranges. Categorical variables are reported as frequencies and percentages. Comparisons of continuous variables between groups were carried out using Wilcoxon-Mann-Whitney test. Comparisons between groups of categorical variables were carried out with chi-square test. Multivariate logistic regression analysis was applied to determine the independent contribution of PAI-1 4G/5G polymorphism on 30-day and 6-month mortality, after controlling for diabetes mellitus, ischemic heart disease, COPD, age, SOFA score and serum lactic acid concentrations. Odds ratio (OR) and 95% confidence intervals (CI) were calculated as measurement of the clinical impact of the predictor variables. We used linear regression modelling to analyze the relationship between plasma PAI-1 concentrations as the dependent variable, and 4G/5G polymorphism, diabetes mellitus, ischemic heart disease and COPD as independent variables. Regression coefficients and 95% CI were calculated as measurement of the clinical impact of the predictor variables. Survival curves at 30 days and 6 months, using 4G/4G vs other genotypes of PAI-1 gen, were plotted using the Kaplan-Meier method and compared by log-rank test. We used Chi-square to test Hardy-Weinberg equilibrium among our genotypes. A P value of less than 0.05 was considered statistically significant. Statistical analyses were performed with SPSS 17.0 (SPSS Inc., Chicago, IL, USA) and NCSS 2000 (Kaysville, Utah).

## Results

We included a total of 260 patients with severe sepsis; 82 (31.5%) with genotype 4G/4G, 126 (48.5%) with genotype 4G/5G and 52 (20.0%) with genotype 5G/5G of the PAI-1 gene. We found no significant deviation from Hardy-Weinberg equilibrium among our genotypes (Chi-square = 0.08; p = 0.78)

As shown in Table 1, there were no significant differences between different genotypes in gender, age, diabetes mellitus, ischemic heart disease, CRF, site of infection, microorganism responsible, bloodstream infection, septic shock, empiric antimicrobial treatment, PaO_2_/FIO_2_, creatinin, bilirubin, leukocytes count, INR, APACHE-II score and SOFA score. However, patients with the 4G/4G genotype showed higher lactatemia, aPTT, and plasma PAI-1 concentrations, and lower platelet count and COPD rate than patients with other genotypes. In addition, patients with the 4G/4G genotype showed higher 30-day and 6-month mortality than patients with other genotypes (Table 2). In addition, we have not found statistically significant differences in 30-day (p = 0.53) and 6-month (p = 0.42) mortality rate between the different ICUs.

**Table 1:** Characteristics of severe septic patients according to genotype in the PAI-1 gene.

	4G/4G (n = 82)	4G/5G (n = 126)	5G/5G (n = 52)	4G/5G more 5G/5G (n = 178)	P-value 4G/4G vs others
Gender female–n (%)	30 (36.6)	44 (34.9)	16 (30.8)	60 (33.7)	0.68
Age—median years (percentile 25–75)	63 (50–74)	60 (48–70)	57 (46–68)	58 (47–69)	0.37
Diabetes Mellitus–n (%)	21 (25.6)	36 (28.6)	16 (30.8)	52 (29.2)	0.66
COPD–n (%)	4 (4.9)	19 (15.1)	8 (15.4)	27 (15.2)	0.02
Ischemic heart disease–n (%)	11 (13.4)	15 (11.9)	4 (7.7)	19 (10.7)	0.54
Site of infection–n (%)					0.52
Respiratory	42 (51.2)	76 (60.3)	26 (50.0)	102 (57.3)	
Abdominal	31 (37.8)	35 (27.8)	12 (23.1)	47 (26.4)	
Neurological	1 (1.2)	1 (0.8)	3 (5.8)	4 (2.2)	
Urinary	4 (4.9)	3 (2.4)	4 (7.7)	7 (3.9)	
Skin	2 (2.4)	5 (4.0)	4 (7.7)	9 (5.1)	
Endocarditis	2 (2.4)	5 (4.0)	3 (5.8)	8 (4.5)	
Osteomyelitis	0	1 (0.8)	0	1 (0.6)	
Microorganism responsibles–n (%)					
Unkwon	44 (53.7)	62 (49.2)	29 (55.8)	91 (51.1)	0.79
Gram-positive	19 (23.2)	35 (27.8)	9 (17.3)	44 (24.7)	0.88
Gram-negative	20 (24.4)	31 (24.6)	12 (23.1)	43 (24.2)	0.99
Fungii	3 (3.7)	1 (0.8)	3 (5.8)	4 (2.2)	0.68
Anaerobe	2 (2.4)	1 (0.8)	0	1 (0.6)	0.24
Bloodstream infection–n (%)	15 (18.3)	17 (13.5)	8 (15.4)	25 (14.0)	0.46
Empiric antimicrobial treatment–n (%)					0.73
Unkown if adequate due to negative cultures	43 (52.4)	63 (50.0)	29 (55.8)	92 (51.7)	
Unkown if adequate due to diagnosis by antigenuria	2 (2.4)	3 (2.4)	1 (1.9)	4 (2.2)	
Adequate	35 (42.7)	52 (41.3)	20 (38.5)	72 (40.4)	
Inadequate	2 (2.4)	8 (6.3)	2 (3.8)	10 (5.6)	
Betalactamic more aminoglycoside–n (%)	18 (22.0)	30 (23.8)	14 (26.9)	44 (24.7)	0.75
Betalactamic more quinolone–n (%)	43 (52.4)	63 (50.0)	29 (55.8)	92 (51.7)	0.99
Septic shock–n (%)	72 (87.8)	105 (83.3)	45 (86.5)	150 (84.3)	0.57
Pa0_2_/FI0_2_ ratio—median (percentile 25–75)	194 (104–279)	183 (118–253)	152 (101–253)	180 (114–250)	0.99
Creatinine (mg/dl)—median (percentile 25–75)	1.30 (0.90–2.20)	1.30 (0.80–2.35)	1.45 (0.80–2.60)	1.40 (0.80–2.40)	0.97
Bilirubin (mg/dl)—median (percentile 25–75)	0.90 (0.50–2.55)	0.88 (0.40–1.64)	1.15 (0.50–1.73)	0.90 (0.44–1.70)	0.62
Leukocytes (cells/mm^3^)—median*10^3^ (percentile 25–75)	14.0 (8.1–22.5)	14.9 (8.6–19.7)	16.0 (10.2–22.3)	15.1 (9.6–20.4)	0.61
Lactic acid (mmol/L)—median (percentile 25–75)	3.20 (1.50–4.65)	1.80 (1.05–4.10)	2.00 (1.18–3.57)	2.00 (1.10–4.00)	0.004
Platelets (cells/mm^3^)—median*10^3^ (percentile 25–75)	156 (82–237)	190 (119–271)	186 (97–255)	190 (117–266)	0.050
INR—median (percentile 25–75)	1.36 (1.11–1.68)	1.29 (1.09–1.51)	1.34 (1.16–1.62)	1.33 (1.10–1.51)	0.29
aPTT (seconds)—median (percentile 25–75)	36 (30–44)	32 (28–44)	32 (27–44)	32 (28–44)	0.03
APACHE-II score- median (percentile 25–75)	21 (17–25)	20 (16–25)	20 (15–24)	20 (15–24)	0.32
SOFA score—median (percentile 25–75)	9 (7–13)	9 (7–12)	10 (8–12)	9 (7–12)	0.34
PAI-1 levels (ng/mL)—median (percentile 25–75)	60.3 (38.5–81.0)	36.2 (20.0–64.7)	28.2 (17.4–63.4)	33.1 (18.6–63.6)	<0.001

COPD = chronic obstructive pulmonary disease; PaO_2_/FIO_2_ = pressure of arterial oxygen/fraction inspired oxygen; INR = International normalized ratio aPTT = Activated partial thromboplastin time; APACHE = Acute Physiology and Chronic Health Evaluation; SOFA = Sepsis-related Organ Failure Assessment.

**Table 2:** Early and late mortality according to genotype in the PAI-1 genetis polymorphis.

	4G/4G (n = 82)	4G/5G (n = 126)	5G/5G (n = 52)	4G/5G or 5G/5G (n = 178)	P-value 4G/4G vs others
30-day mortality—n (%)	37 (45.1)	36 (28.6)	16 (30.8)	52 (29.2)	0.02
6-month mortality—n (%)	45 (54.9)	43 (34.1)	21 (40.4)	64 (35.9)	0.01

Multiple logistic regression analysis showed that the 4G/4G genotype was associated with higher mortality at 30 days (Odds Ratio = 1.95; 95% CI = 1.063–3.561; p = 0.03) and at 6 months (Odds Ratio = 2.19; 95% CI = 1.221–3.934; p = 0.01), after controlling for diabetes mellitus, ischemic heart disease, COPD, age, SOFA score and serum lactic acid concentrations (Tables 3 and 4). In addition, multiple logistic regression analysis showed that plasma PAI-1 concentrations were associated with higher mortality at 30 days (Odds Ratio = 1.01; 95% CI = 1.002–1.022; p = 0.02) and at 6 months (Odds Ratio = 1.01; 95% CI = 1.003–1.023; p = 0.01), after controlling for diabetes mellitus, ischemic heart disease, COPD, age, SOFA score and serum lactic acid concentrations (Tables 3 and 4).

**Table 3:** Multiple logistic regression analyses to predict 30-day mortality.

	Odds Ratio	95% Confidence Interval	*P*-value
**FIRST MODEL**			
Genotype of PAI-1 gene (4G/4G vs other)	1.95	1.063–3.561	0.03
Diabetes Mellitus (yes vs non)	2.16	1.160–4.028	0.02
Chronic obstructive pulmonary disease (yes vs non)	1.07	0.434–2.649	0.88
Ischemic heart disease (yes vs non)	0.56	0.220–1.440	0.23
SOFA (points)	1.15	1.056–1.249	0.001
Lactic acid levels (mmol/L)	1.13	1.020–1.247	0.02
Age (years)	1.02	0.998–1.039	0.08
**SECOND MODEL**			
Plasma PAI-1 levels (ng/mL)	1.01	1.002–1.022	0.02
Diabetes Mellitus (yes vs non)	2.05	1.097–3.835	0.02
Chronic obstructive pulmonary disease (yes vs non)	0.94	0.385–2.311	0.90
Ischemic heart disease (yes vs non)	0.62	0.244–1.578	0.32
SOFA (points)	1.14	1.044–1.236	0.003
Lactic acid levels (mmol/L)	1.09	0.984–1.211	0.10
Age (years)	1.02	0.997–1.038	0.10

**Table 4:** Multiple logistic regression analyses to predict 6-month mortality.

	Odds Ratio	95% Confidence Interval	*P*-value
**FIRST MODEL**			
Genotype of PAI-1 gene (4G/4G vs other)	2.19	1.221–3.934	0.01
Diabetes Mellitus (yes vs non)	1.88	1.025–3.437	0.04
Chronic obstructive pulmonary disease (yes vs non)	1.70	0.726–3.969	0.22
Ischemic heart disease (yes vs non)	0.75	0.314–1.794	0.52
SOFA (points)	1.11	1.027–1.207	0.01
Lactic acid levels (mmol/L)	1.15	1.035–1.278	0.01
Age (years)	1.01	0.994–1.033	0.18
**SECOND MODEL**			
Plasma PAI-1 levels (ng/mL)	1.01	1.003–1.023	0.01
Diabetes Mellitus (yes vs non)	1.78	0.969–3.270	0.06
Chronic obstructive pulmonary disease (yes vs non)	1.47	0.636–3.390	0.37
Ischemic heart disease (yes vs non)	0.83	0.351–1.966	0.67
SOFA (points)	1.10	1.015–1.192	0.02
Lactic acid levels (mmol/L)	1.11	0.994–1.234	0.06
Age (years)	1.01	0.993–1.032	0.20

Multivariate linear regression analysis showed that plasma PAI-1 concentrations were associated with PAI-1 4G/5G polymorphism, after controlling for diabetes mellitus, ischemic heart disease and COPD (regression coefficient = 4.82; 95% CI = 3.227 to 6.406; p<0.001) (Table 5).

**Table 5:** Lineal multivariate regression analysis to predict plasma PAI-1 levels.

	Regression coefficient	95% Confidence Interval	*P*-value
Genotype of PAI-1 gene (4G/4G vs other)	4.82	3.227 to 6.406	<0.001
Diabetes Mellitus	4.84	-3.410 to 13.090	0.25
Chronic obstructive pulmonary disease	0.53	-10.951 to 12.005	0.93
Ischemic heart disease	2.52	-14.208 to 9.178	0.67

**Fig 1:**
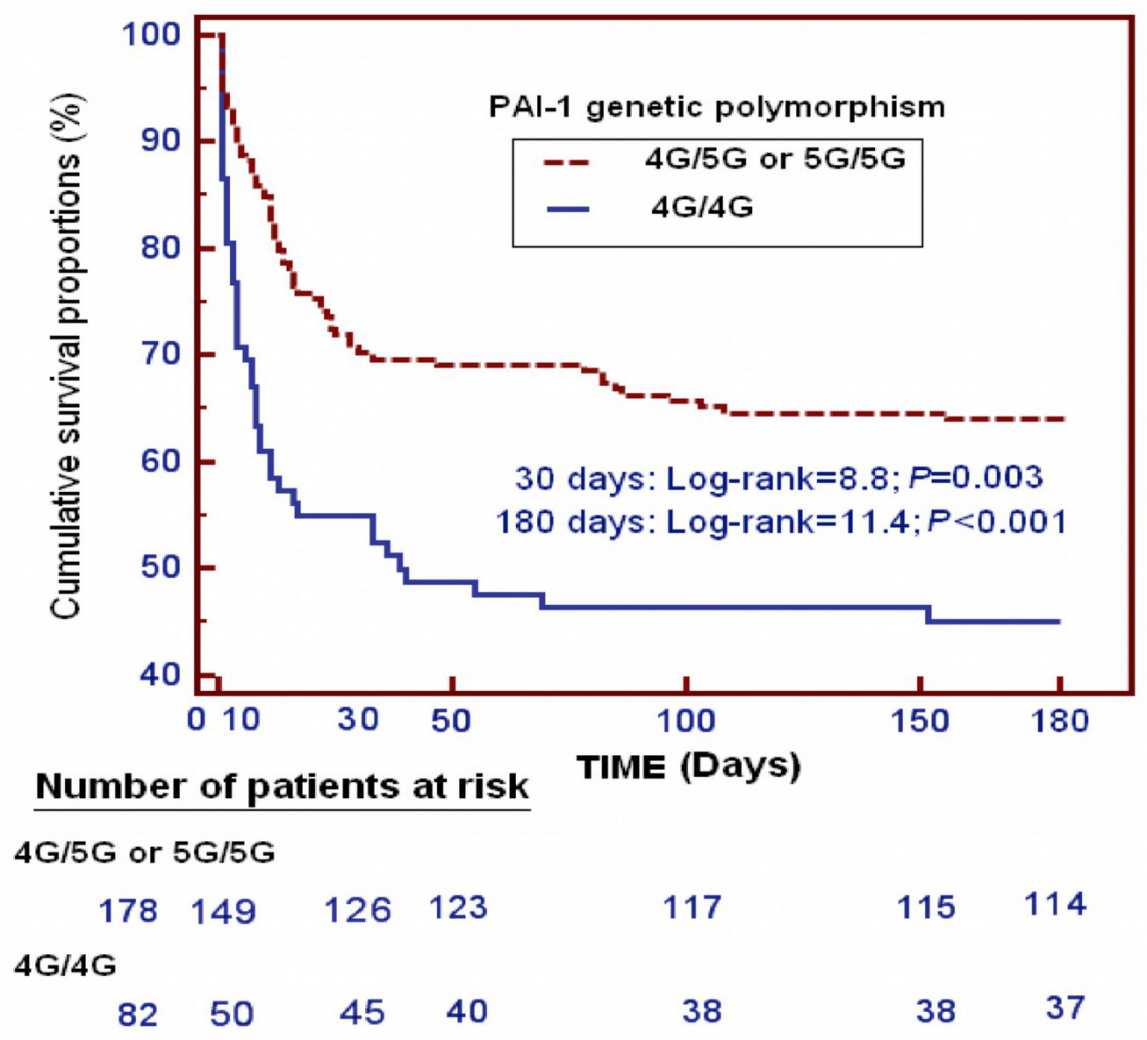
Kaplan-Meier curves showing the cumulative proportion of survival patients at 30 days and 6 months according to the presence of 4G/4G vs other genotypes of PAI-1 genetic polymorphism.

Survival analysis showed that patients with the PAI-1 4G/4G genotype presented lower 30-day survival (Chi-square = 8.82; Hazard ratio = 1.9 (95% CI = 1.17–2.95); p = 0.003) and lower 6-month survival (Chi-square = 8.82; p = 0.003) than those with other genotypes (Chi-square = 11.4; Hazard ratio = 1.9 (95% CI = 1.24–2.88); p<0.001) (Fig 1).

## Discussion

To our knowledge, this is the largest study to date reporting data about PAI-1 4G/5G polymorphism, plasma PAI-1 concentrations and mortality in septic patients. The major findings of our study were that septic patients with the 4G/4G genotype had higher plasma PAI-1 concentrations and higher risk of death than those with 4G/5G or 5G/5G genotype.

Previous studies have found higher plasma PAI-1 concentrations in septic patients than in controls [25–28], and higher plasma PAI-1 concentrations in non-surviving than in surviving septic patients [3–12]. In addition, patients with the PAI-1 4G/4G genotype had a higher risk of death than patients with the 4G/5G or 5G/5G genotypes, according to a recently published meta-analysis [21]. In one study involving 175 children with meningococcal disease [10] and another with 88 adult septic shock patients [11], it was found that patients with the 4G/4G genotype of the PAI-1 gene had higher plasma PAI-1 concentrations and higher risk of death than those with the 4G/5G or 5G/5G genotypes. In addition, another study that included 166 adult patients with sepsis reported that patients with the 4G/4G genotype and those with higher plasma PAI-1 concentrations had a higher risk of death; however, the association between PAI-1 4G/5G polymorphism and plasma PAI-1 concentrations was not reported [12]. García-Segarra et al [11] and Wingeyer et al [12] carried out a regression analysis to determine the independent contribution of PAI-1 4G/5G polymorphism on 30-day mortality; but this was not done in the study by Hermans et al [10]. In our study, patients with the PAI-1 4G/4G genotype showed higher mortality at 30 days and at 6 months on multiple logistic regression analysis, and that plasma PAI-1 concentrations were associated with the PAI-1 4G/5G polymorphism on linear regression analysis (not reported in previous studies).

The frequencies of the PAI-1 genotypes in our septic patients (31.5% with 4G/4G, 48.5% with 4G/5G and 20.0% with 5G/5G genotype) are similar to those found in the meta-analysis by Li et al (30.8% with 4G/4G, 47.2% with 4G/5G and 22.0% with the 5G/5G genotype) [21].

During sepsis there is coagulation activation and fibrinolysis inhibition, contributing to capillary thrombosis, multiple organ dysfunction, and finally death. PAI-1 is a member of the serine protease inhibitor (serpin) family, which regulates fibrinolysis by inhibiting the tissue-type plasminogen activator (t-PA) and the urokinase-type plasminogen activator (u-PA) [2]. PAI-1 plays an important role in the down-regulation of fibrinolysis during sepsis [29]. Consequently, high concentrations of PAI-1 contribute to multiple organ dysfunction, and thus, increase the risk of death. We believe that the higher mortality observed in patients with the 4G/4G genotype was related with their higher plasma PAI-1 concentrations, and this may have favoured the appearance of capillary thrombosis, which in turn could have led to multiple organ dysfunction, and finally death.

Therefore, a possible approach for the treatment of severe sepsis could be the control of PAI-1 activity in order to modulate fibrinolysis to avoid organ dysfunction and thus decrease the mortality rate. Some strategies have also been found to inhibit the PAI-1 activity in *in vitro* models [30–35] and in animal models [36–38]. However, to date there are no studies reporting the clinical application of PAI-1 inhibitors in septic patients. Thus, more research is necessary before the inhibition of PAI-1 can be considered a novel therapy for septic patients.

We think that the determination of the PAI-1 4G/5G polymorphism could help in the selection of patients who have more risk of death. The strengths of our study are the large sample size (n = 260), the fact that it was a multicenter study, and that we carried out linear regression modelling to analyze the relationship between plasma PAI-1 concentrations and 4G/5G polymorphism, after controlling for other variables. On the other hand, the study has certain limitations. First, we did not determine PAI-1 genetic polymorphisms in healthy control subjects and in non-septic critically ill patients; however, the objective of our study was not to determine the association between the polymorphism and the occurrence of sepsis, but rather the association between the polymorphism and sepsis survival. Second, we did not analyse other genes and a gene-gene interaction is possible [39,40]. Thus, although the 4G/4G genotype seems to be associated with increased plasma PAI-1 concentrations and mortality, new studies with large sample sizes are needed to determine whether there is some gene-gene interaction that affects the prognosis of septic patients. Third, most of our patients were from the Canary Islands and the results may not be generalizable to other populations. Fourth, we found that carriage of the PAI-1 4G/4G genotype was associated with higher mortality after controlling for diabetes mellitus, ischemic heart disease, COPD, age, SOFA score and serum lactic acid concentrations; however other factors could have played a role in this.

## Conclusions

To our knowledge, this is the largest study to date reporting data about PAI-1 4G/5G polymorphism, plasma PAI-1 concentrations and mortality in septic patients. The major findings of our study were that septic patients with the 4G/4G genotype had higher plasma PAI-1 concentrations and higher risk of early death than those with the 4G/5G or 5G/5G genotype.
